# Supplementary material for: Analog quantum simulation of the Rabi model in the ultra-strong coupling regime
Source: Nat Commun. 2017 Oct 3;8:779. doi: 10.1038/s41467-017-00894-w (PMC5626763; doi:10.1038/s41467-017-00894-w)
Supplement: Supplementary file 1 — Supplementary Information [file 41467_2017_894_MOESM1_ESM.pdf]

Supplementary Note 1. THEORETICAL BASICS OF THE SIMULATION SCHEME

The simulation scheme we detail here follows a proposal published in Ref. [1]. The quantum Rabi Hamiltonian to be constructed reads

$$\frac{\hat{H}}{\hbar} = \frac{\epsilon}{2}\hat{\sigma}_z + \omega\hat{b}^\dagger\hat{b} + g\hat{\sigma}_x\left(\hat{b}^\dagger + \hat{b}\right), \tag{1}$$

with ϵ the qubit energy splitting, ω the resonator frequency and g the transversal coupling strength. $\hat{\sigma}_i$ are Pauli matrices with $\hat{\sigma}_z\left|\mathrm{g}\right\rangle = \left|\mathrm{g}\right\rangle$ and $\hat{\sigma}_z\left|\mathrm{e}\right\rangle = -\left|\mathrm{e}\right\rangle$, where $\left|\mathrm{g}\right\rangle$, $\left|\mathrm{e}\right\rangle$ are the qubit groundstate and first excited state, respectively. We neglect the qubit tunneling matrix element Δ such that the qubit Hamiltonian remains

$$\frac{\hat{H}_\mathrm{q}}{\hbar} = \frac{\epsilon}{2}\hat{\sigma}_z + \frac{\Delta}{2}\hat{\sigma}_x \approx \frac{\epsilon}{2}\hat{\sigma}_z. \tag{2}$$

$\Delta \ll \epsilon$ is valid for the transmon qubit since hopping between wells of the Josephson potential is suppressed in the spirit of a WKB approximation [2]. $\hat{b}^\dagger$ ($\hat{b}$) are creation (annihilation) operators in the resonator Fock space. Throughout this derivation, operators acting on either the qubit or the resonator subspace of the joint Hilbert space are written without the formally correct tensor product with the unity in the other subspace, such that $\mathbb{1}_2 \otimes \hat{b}^\dagger\hat{b} \equiv \hat{b}^\dagger\hat{b}$, $\hat{\sigma}_z \otimes \mathbb{1}_\mathrm{r} \equiv \hat{\sigma}_z$. $\mathbb{1}_2$ denotes unity in the qubit Hilbert space while $\mathbb{1}_\mathrm{r}$ denotes unity in the bosonic mode Hilbert space of dimension r. Likewise, the tensor product symbol is omitted for clarity.

The qubit and the bosonic oscillator mode are physical elements of the quantum simulator implemented on chip. Since the geometric coupling g on chip is small compared to the mode energies, $g/\epsilon \ll 1$ and $g/\omega \ll 1$, the rotating wave approximation (RWA) is valid and Supplementary Equation (1) takes the form of the Jaynes-Cummings Hamiltonian [3]. The construction of the effective Hamiltonian relies on the application of two transversal microwave tones acting on the qubit. The key feature is a renormalization of the mode energies ϵ, ω by experimental parameters in the frame rotating with the first dominant drive. Even though a RWA may be used to simplify the ordinary Jaynes-Cummings Hamiltonian as implemented on chip, the RWA breaks down for the quantum Rabi Hamiltonian in the ultra-strong coupling (USC) regime and beyond. Therefore the complete coupling term $g\hat{\sigma}_x(\hat{b}^\dagger + \hat{b})$ needs to be preserved. The Jaynes-Cummings Hamiltonian in the laboratory frame with the two drives applied takes the form

$$\frac{\hat{H}}{\hbar} = \frac{\epsilon}{2}\hat{\sigma}_z + \omega\hat{b}^\dagger\hat{b} + g\left(\hat{\sigma}_-\hat{b}^\dagger + \hat{\sigma}_+\hat{b}\right) + \hat{\sigma}_x\eta_1\cos(\omega_1 t + \varphi_1) + \hat{\sigma}_x\eta_2\cos(\omega_2 t + \varphi_2),$$

with η_i the amplitudes, ω_i the frequencies and φ_i the relative phase of drive i. In the following derivation we set $\varphi_i = 0$ without loss of generality. We use Pauli's ladder operators $\hat{\sigma}_\pm = 1/2\left(\hat{\sigma}_x \pm \mathrm{i}\hat{\sigma}_y\right)$. The unitary transformation $\hat{U}$ for changing into the frame rotating with the dominant drive η_1 reads

$$\hat{U} = \exp\left\{\mathrm{i}\omega_1 t\left(\hat{b}^\dagger\hat{b} + \frac{1}{2}\hat{\sigma}_z\right)\right\}. \tag{3}$$

Performing the transformation $|\tilde{\psi}\rangle = U|\psi\rangle$ of eigenstates $|\psi\rangle$ leads to a transformed Hamiltonian $\tilde{H}$ according to

$$\tilde{H} = \hat{U}\hat{H}\hat{U}^\dagger - \mathrm{i}\hat{U}\dot{\hat{U}}^\dagger. \tag{4}$$

The transformation of Supplementary Equation (3) yields

$$\begin{aligned}
\frac{\tilde{H}}{\hbar} =& \frac{\epsilon}{2}\hat{\sigma}_z + \omega\hat{b}^\dagger\hat{b} + g\left(\hat{\sigma}_+e^{\mathrm{i}\omega_1 t} + \hat{\sigma}_-^{-\mathrm{i}\omega_1 t}\right)\left(e^{\mathrm{i}\omega_1 t}\hat{b}^\dagger + e^{-\mathrm{i}\omega_1 t}\hat{b}\right) \\
&+ \eta_1\left(\hat{\sigma}_+e^{\mathrm{i}\omega_1 t} + \hat{\sigma}_-e^{-\mathrm{i}\omega_1 t}\right)\cos\omega_1 t + \eta_2\left(\hat{\sigma}_+e^{\mathrm{i}\omega_1 t} + \hat{\sigma}_-e^{-\mathrm{i}\omega_1 t}\right)\cos\omega_2 t - \omega_1\left(\hat{b}^\dagger\hat{b} + \frac{1}{2}\hat{\sigma}_z\right) \qquad (5) \\
=& \frac{1}{2}\left(\epsilon - \omega_1\right)\hat{\sigma}_z + \left(\omega - \omega_1\right)\hat{b}^\dagger\hat{b} + g\left(\hat{\sigma}_-\hat{b}^\dagger + \hat{\sigma}_+\hat{b}\right) + \frac{\eta_1}{2}\left(\hat{\sigma}_+ + \hat{\sigma}_-\right) + \frac{\eta_2}{2}\left(\hat{\sigma}_+e^{\mathrm{i}(\omega_1-\omega_2)t} + \hat{\sigma}_-e^{-\mathrm{i}(\omega_1-\omega_2)t}\right) \\
&+ g\left(\hat{\sigma}_+e^{2\mathrm{i}\omega_1 t}\hat{b}^\dagger + \hat{\sigma}_-e^{-2\mathrm{i}\omega_1 t}\hat{b}\right) + \frac{\eta_1}{2}\left(\hat{\sigma}_+e^{2\mathrm{i}\omega_1 t} + \hat{\sigma}_-e^{-2\mathrm{i}\omega_1 t}\right) + \frac{\eta_2}{2}\left(\hat{\sigma}_+e^{\mathrm{i}(\omega_1+\omega_2)t} + \hat{\sigma}_-e^{\mathrm{i}(-\omega_1-\omega_2)t}\right). \qquad (6)
\end{aligned}$$

Terms of the form $e^X Y e^{-X}$ are calculated using the power series expansion of the exponential function, also called Hadamard lemma. For $X = \mathrm{i}\omega_1 t\hat{b}^\dagger\hat{b}$, $Y = \hat{b}^\dagger$,

$$e^{\mathrm{i}\omega_1 t\hat{b}^\dagger\hat{b}}\hat{b}^\dagger e^{-\mathrm{i}\omega_1 t\hat{b}^\dagger\hat{b}} = e^{\mathrm{i}\omega_1 t}\hat{b}^\dagger \tag{7}$$

$$e^{\mathrm{i}\omega_1 t\hat{b}^\dagger\hat{b}}\hat{b}e^{-\mathrm{i}\omega_1 t\hat{b}^\dagger\hat{b}} = e^{-\mathrm{i}\omega_1 t}\hat{b} \tag{8}$$

since

$$\sum_{m=0}^{\infty}\frac{1}{m!}\left[\mathrm{i}\omega_1 t\hat{b}^\dagger\hat{b},\hat{b}^\dagger\right]_m=\hat{b}^\dagger\sum_{m=0}^{\infty}\frac{1}{m!}\left(\mathrm{i}\omega_1 t\right)^m=\hat{b}^\dagger e^{\mathrm{i}\omega_1 t}. \tag{9}$$

$e^{\frac{\mathrm{i}}{2}\omega_1 t\hat{\sigma}_z}$ and $\hat{\sigma}_z$ clearly commute, while

$$e^{\frac{\mathrm{i}}{2}\omega_1 t\hat{\sigma}_z}\hat{\sigma}_x e^{-\frac{\mathrm{i}}{2}\omega_1 t\hat{\sigma}_z}=\hat{\sigma}_+e^{\mathrm{i}\omega_1 t}+\hat{\sigma}_-e^{-\mathrm{i}\omega_1 t} \tag{10}$$

using $e^{\mathrm{diag}(a,b)}=\mathrm{diag}(e^a,e^b)$. Terms in the last line in Supplementary Equation (6) are omitted in the following within the RWA, valid for $\eta_1/2\omega_1\ll 1$. This is a good approximation as η_1 is bound in the experiment by the qubit anharmonicity $|\alpha|\lesssim 350\,\mathrm{MHz}$.

The η_1 term in the transformed Hamiltonian Supplementary Equation (6) is the most significant term, which justifies to move to its interaction picture. Under the constraint $\eta_1\equiv\omega_1-\omega_2$, the Hamiltonian in the interaction picture becomes

$$\begin{aligned}e^{\mathrm{i}\frac{\eta_1}{2}\hat{\sigma}_x t}\left[\frac{\tilde{H}}{\hbar}-\frac{\eta_1}{2}\hat{\sigma}_x\right]e^{-\mathrm{i}\frac{\eta_1}{2}\hat{\sigma}_x t}=&\frac{\eta_2}{2}\begin{pmatrix}\sin^2\eta_1 t & \cos\eta_1 t+\mathrm{i}\sin 2\eta_1 t\\ \cos\eta_1 t-\mathrm{i}\sin 2\eta_1 t & -\sin^2\eta_1 t\end{pmatrix}\\&+\frac{1}{2}\left(\epsilon-\omega_1\right)\begin{pmatrix}\cos\eta_1 t & -\mathrm{i}\sin\eta_1 t\\ \mathrm{i}\sin\eta_1 t & -\cos\eta_1 t\end{pmatrix}+\left(\omega-\omega_1\right)\hat{b}^\dagger\hat{b}\\&+g\left[\begin{pmatrix}-\frac{1}{2}\mathrm{i}\sin\eta_1 t & \frac{1}{2}(1+\cos\eta_1 t)\\ \frac{1}{2}(1-\cos\eta_1 t) & \frac{1}{2}\mathrm{i}\sin\eta_1 t\end{pmatrix}\hat{b}^\dagger+\begin{pmatrix}\frac{1}{2}\mathrm{i}\sin\eta_1 t & \frac{1}{2}(1-\cos\eta_1 t)\\ \frac{1}{2}(1+\cos\eta_1 t) & -\frac{1}{2}\mathrm{i}\sin\eta_1 t\end{pmatrix}\hat{b}\right].\end{aligned} \tag{11}$$

Performing a time averaging in the spirit of a RWA casts Supplementary Equation (11) into the desired form of the quantum Rabi Hamiltonian

$$\frac{\hat{H}_{\mathrm{eff}}}{\hbar}=\frac{\eta_2}{2}\frac{\hat{\sigma}_z}{2}+\omega_{\mathrm{eff}}\hat{b}^\dagger\hat{b}+\frac{g}{2}\sigma_x\left(\hat{b}^\dagger+\hat{b}\right), \tag{12}$$

with $\omega_{\mathrm{eff}}\equiv\omega-\omega_1$ and noting that $\eta_1\gg\eta_2$.

In the experiment, the applied Rabi drives parasitically couple to the bosonic mode since the elements are degenerate during the simulation experiment and spatially close by in the circuit. This effect is accounted for by an additional term

$$\eta_{\mathrm{r}}\left(\hat{b}^\dagger+\hat{b}\right)\cos(\omega_1 t) \tag{13}$$

in the laboratory frame Hamiltonian, Supplementary Equation (3). η_{r} denotes the effective amplitude of the parasitic drive and we take into account the effect of only the dominant Rabi drive frequency ω_1. Performing the transformation $\hat{U}$ yields

$$\frac{\eta_{\mathrm{r}}}{2}\left(\hat{b}^\dagger+\hat{b}\right)+\frac{\eta_{\mathrm{r}}}{2}\left(e^{2\mathrm{i}\omega_1 t}\hat{b}^\dagger+e^{-2\mathrm{i}\omega_1 t}\hat{b}\right). \tag{14}$$

Terms rotating with $e^{\pm 2\mathrm{i}\omega_1 t}$ ore omitted, resulting in a time independent drive term that is added to the Hamiltonian (12) in the rotating frame. The effective Hamiltonian including the parasitic drive term reads

$$\frac{\hat{H}_{\mathrm{eff,p}}}{\hbar}=\frac{\eta_2}{2}\frac{\hat{\sigma}_z}{2}+\omega_{\mathrm{eff}}\hat{b}^\dagger\hat{b}+\frac{g}{2}\sigma_x\left(\hat{b}^\dagger+\hat{b}\right)+\frac{\eta_{\mathrm{r}}}{2}\left(\hat{b}^\dagger+\hat{b}\right). \tag{15}$$

By applying the unitary displacement transformation

$$\hat{D}=\exp\left\{-\frac{\eta_{\mathrm{r}}}{2\omega_{\mathrm{eff}}}\left(\hat{b}^\dagger-\hat{b}\right)\right\} \tag{16}$$

we can cast the Hamiltonian (15) in the original form of the quantum Rabi Hamiltonian, including a qubit tunneling term $\propto\hat{\sigma}_x$,

$$\hat{D}^\dagger\frac{\hat{H}}{\hbar}\hat{D}=\frac{\eta_2}{2}\frac{\hat{\sigma}_z}{2}+\omega_{\mathrm{eff}}\left(\hat{b}^\dagger-\frac{\eta_{\mathrm{r}}}{2\omega_{\mathrm{eff}}}\right)\left(\hat{b}-\frac{\eta_{\mathrm{r}}}{2\omega_{\mathrm{eff}}}\right)+\frac{g}{2}\sigma_x\left(\hat{b}^\dagger+\hat{b}-\frac{\eta_{\mathrm{r}}}{\omega_{\mathrm{eff}}}\right)+\frac{\eta_{\mathrm{r}}}{2}\left(\hat{b}^\dagger+\hat{b}-\frac{\eta_{\mathrm{r}}}{2\omega_{\mathrm{eff}}}\right) \tag{17}$$

$$=\frac{\eta_2}{2}\frac{\hat{\sigma}_z}{2}-g\frac{\eta_{\mathrm{r}}}{2\omega_{\mathrm{eff}}}\hat{\sigma}_x+\omega_{\mathrm{eff}}\hat{b}^\dagger\hat{b}+\frac{g}{2}\sigma_x\left(\hat{b}^\dagger+\hat{b}\right)+\mathrm{const.}, \tag{18}$$

using

$$\hat{D}^\dagger \hat{b}^\dagger \hat{b} \hat{D} = \hat{D}^\dagger \hat{b}^\dagger \hat{D} \hat{D}^\dagger \hat{b} \hat{D} = \left(\hat{b}^\dagger - \frac{\eta_\mathrm{r}}{2\omega_\mathrm{eff}} \right) \left(\hat{b} - \frac{\eta_\mathrm{r}}{2\omega_\mathrm{eff}} \right). \tag{19}$$

Supplementary Note 2. SAMPLE FABRICATION

Sample fabrication was carried out in one single electron beam lithography step. The Josephson junctions are formed by shadow angle evaporation and a Dolan bridge technique with electrode film thicknesses of 30 nm and 50 nm, respectively, resulting in a Al film thickness of 80 nm across the entire chip. The area of the Josephson junctions is designed to be 100 nm × 220 nm, resulting in a critical current $I_\mathrm{c} = 45\,\mathrm{nA}$ for a single junction. Al is evaporated at a chamber background pressure of about 3×10^{-8} mbar. We applied an Al metalization on the backside of the double-side polished intrinsic Si substrate as a ground reference for the microstrip elements on chip. Electric field coupling in the substrate due to the backside metalization accounts for roughly half of the qubit capacitance [4].

Supplementary Note 3. EXPERIMENTAL SETUP

A. Microwave setup

The schematic diagram of the measurement and microwave setup is depicted in Supplementary Figure 1. We readout the qubit state by observing a dispersive shift of the readout resonator which is acquired via a 400 ns long readout pulse. The resonator shift is extracted from the microwave reflection signal at a single-ended 50 Ω matched transmission line that capacitively couples to the readout resonator. We frequency-convert the readout pulse by heterodyne single sideband mixing to eliminate parasitic population of the readout device during pulse-off time.

Qubit excitation and Rabi driving are performed by heterodyne mixing with respective IQ frequencies for different drive and excitation frequencies, using one single microwave source and one IQ mixer, see Supplementary Figure 1(a). This allows for the required phase control on the phases φ_1, φ_2 of the Rabi drives. In particular, we fix the idling time between initial excitation pulse and the onset of the Rabi drive, such that the acquired phase during that time is constant, and apply the Rabi drives with a constant relative phase φ_i with respect to the phase used for the excitation pulse. We chose an LO frequency located 20 MHz or 65 MHz above the qubit control frequency, located at about $\omega/2\pi + 95\,\mathrm{MHz}$. In the experiment with the second Rabi drive added, we generate the drives initially in separate IQ mixers that share a common LO input. We suppress phase errors by employing identical coaxial cables for the high-frequency lines prior to the combination of the drive pulses. The microwave pulses for $\hat{X}\hat{Y}$ control of the qubit are applied via the same transmission line used for readout.

The qubit transition frequency is adjusted by a dc current applied to the on-chip flux coil. High frequency noise is filtered at the 4 K stage with RCR type π-filters at about 25 kHz and on the base plate via an RC-element enclosed in copper powder [5]. Fast flux pulses for fast $\hat{Z}$ pulsing of the qubit are sent through a separate microwave line and combined with the offset current by means of a bias tee located at the base plate.

B. Compensation for finite bias tee time constant

In order to combine dc and ac flux signals that are applied to the flux bias line without breaking the 50 Ω impedance matching, we make use of a bias tee in the experiment. Due to a finite time constant τ of the bias tee, a continuous compensation for decharging effects is required in order to produce the desired pulse sequence in the flux bias line on chip. Supplementary Figure 2(a) shows the schematic circuit diagram present during flux pulse generation. V_ex denotes the amplitude of the voltage pulse, R is the line resistance in front of the bias tee and C is the relevant capacitance of the bias tee. From Kirchhoff's law we can write

$$RI + \frac{Q}{C} = V_\mathrm{ex}, \tag{20}$$

with I the current that is admitted to the flux bias line. The condition of constant current follows from requesting

$$\frac{\mathrm{dI}}{\mathrm{dt}} = \frac{1}{R}\frac{\mathrm{d}}{\mathrm{dt}} V_\mathrm{ex} - \frac{1}{RC} I \overset{!}{=} 0, \tag{21}$$

which yields

$$\frac{\mathrm{d}}{\mathrm{dt}}V_{\mathrm{ex}} = \frac{I}{C}. \tag{22}$$

Since I is not a function of time according to the initial claim, Supplementary Equation (22) can be integrated yielding

$$V_{\mathrm{ex}} = \frac{1}{C}It + \mathrm{const.} \propto t. \tag{23}$$

This shows that the required correction of the externally applied voltage V_{ex} is linear in time t with a slope proportional to $1/\tau$. See the pulse sequence applied (blue) and the resulting pattern (red) in Supplementary Figure 2(b). During pulse-off time, V_{ex} has to be kept constant and no further correction is required. The described compensation cannot be performed for longer than approximately 1 µs within one continuous pulse sequence, since the output stage of our pulse generator eventually saturates. With additional amplification of the generated signal, the current is increasing linearly during compensation time, implying an experimental limitation as higher currents can ultimately heat the cryostat. The utilized pulse sequence therefore scales nicely for perspective longer simulation times Δt since a compensation during Δt is not required.

We calibrated the time constant of the bias tee used in the experiment to be $\tau = 0.7$ µs.

C. Summary of the device parameters

Supplementary Table 1 lists the relevant parameters of the simulation device that were found experimentally and used in the master equation simulations, together with drive parameters typically used for the quantum simulation.

Values for the geometric coupling strength g are summarized in Supplementary Table 2. Values from the spectroscopy experiment and the vacuum Rabi experiment are in reasonable agreement. The geometric coupling appears to be effectively enhanced during the simulation experiment, which could be due to a parameter drift during successive cool-downs. In order to achieve a better agreement between experiment and master equation simulation, we use a slightly increased value for the geometric coupling strength in classical simulations. We believe that the effective modification of this parameter is a consequence of the discussed parasitic driving of the bosonic mode in the experiment. We checked that this has no qualitative influence on the results, in particular the non-conservation of the total excitation number remains evident.

Supplementary Note 4. CALIBRATION OF THE QUBIT BASIS AND BASELINE SHIFT

A. Calibration of the qubit basis

Prior to the experiments presented in the main text, we calibrated the qubit basis spanned by eigenstates $|\mathrm{g}\rangle$, $|\mathrm{e}\rangle$. This is of relevance for the data analysis detailed in Supplementary Note 5 B and to demonstrate the base line shifts observed in Fig. 3 in the main text. The calibration is performed by Rabi spectroscopy including the fast z pulsing scheme as adopted in successive quantum simulation experiments. The applied pulse sequence and measured Rabi oscillations are depicted in Supplementary Figure 3. The length of the applied excitation pulse is given on the horizontal axis in Supplementary Figure 3(b). Fundamental qubit states (black lines) can be mapped to dispersive shifts of the readout resonator with the bosonic mode in its groundstate and off-resonant.

B. Qubit basis shift

The qubit state is expected to assume an incoherent steady state on the equator of the Bloch sphere for long simulation times Δt due to the finite coherence in our circuit. However, we notice a shift in the steady state qubit population dependent on the initially prepared qubit state. This is apparent in Supplementary Figure 3(c), showing measurements for the qubit prepared in $|\mathrm{e}\rangle$ (blue) and $|\mathrm{g}\rangle$ (green) and in Fig. 3(f)-(i) in the main text. We attribute this effect to a change in the effective qubit basis, which is not captured by the master equation simulations performed. We additionally conjecture that the basis shifts are caused by an effective tilt of the qubit Bloch sphere as an artifact of the frequency tuning in experiment prior to applying the Rabi drives. We isolate the effect as an initialization issue since the basis shift cancels out in good approximation when averaging the mutually antiparallel simulation sequences in Fig. 3(f)-(i).

Supplementary Note 5. CLASSICAL MASTER EQUATION SIMULATION

Numerical simulations are based on a master equation solver provided by the QuTiP package [6, 7] for python. The time evolution of a given initial state or density matrix is calculated by solving the von Neumann equation associated with the given system Hamiltonian in the absence of dissipation. For including losses to an imperfect environment of the system Hamiltonian, the time evolution of the density matrix is calculated via the Lindblad master equation.

Classical simulations in the main text include a finite lifetime of the qubit of about 5 µs, a dephasing time of about 0.5 µs and a bosonic mode inverse lifetime of $\kappa = 3.9 \times 10^6\,\mathrm{s}^{-1}$. The Fock space of the bosonic mode is truncated at a photon number of 25, since higher excitation numbers were found to not play a significant role. The transmon qubit is treated as a three-level system with the experimentally found anharmonicity $\alpha/2\pi = -350\,\mathrm{MHz}$. We use a transversal qubit coupling operator

$$\sum_{ij} \hat{g}^x_{ij} \equiv \sum_{ij} \frac{g_{ij}}{g_{01}} \left|i\right\rangle \left\langle j\right| , \tag{24}$$

with the coupling matrix elements g_{ij} found by evaluating the Cooper pair number operator in the charge basis [8, 9].

A. Verification of the simulation scheme

Since a rotating frame is not an inertial system, the laws of physics may be drastically altered when describing a physical system in a rotating frame. In the framework of analog quantum simulation, this offers a rich toolbox which allows to access intriguing effective parameter regimes that are hard or impossible to access in the laboratory frame. While the qubit dynamics in the rotating frame is well reproduced as demonstrated in Fig. 3 in the main text, it is not a priori clear that the bosonic mode population evolution of the quantum Rabi model is well reflected by the laboratory frame simulation. To verify the simulation scheme for the ideal system we compare the bosonic mode population in the driven laboratory frame, and the expected evolution of the ideal quantum Rabi model via classical master equation simulations. Here, we neglect dissipation and parasitic driving of the bosonic mode. Supplementary Figure 4(a) demonstrates good agreement when comparing the time evolutions of the ideal (solid line) and the constructed (dashed line) Hamiltonians. The violation of excitation number conservation in the quantum Rabi model manifests in excitation numbers of the bosonic mode of larger than one for small ω_{eff}. We find that the maximum photon excitation in the bosonic mode roughly equals one for choosing simulation conditions where $\omega_{\mathrm{eff}} \approx g \sim 2\pi \times 5\,\mathrm{MHz}$.

For $\omega_{\mathrm{eff}}/2\pi = 5\,\mathrm{MHz}$ we demonstrate that the photon population in the bosonic mode is independent of the applied drive amplitude η_1, reflecting the fact that it does not appear in the synthesized Hamiltonian, Supplementary Equation (12). Simulations for various η_1 are depicted in Supplementary Figure 4(b).

B. Qubit population retrieval from measured dispersive shifts of the readout resonator

In measuring the dispersive shift of the readout resonator during the quantum simulation experiment, we observe a bulged and shifted equatorial baseline following the expected population evolution of the bosonic mode as obtained from the master equation simulation. We attribute this to a photon exchange coupling f between the bosonic mode and the readout resonator, potentially mediated by the qubit. By inheriting nonlinearity from the qubit it gives rise to a dispersive shift on the readout resonator dependent on the photon number in the bosonic mode. The complete Hamiltonian including the readout resonator of resonance frequency ω_{r} with creation (annihilation) operator $\hat{a}^\dagger$ $(\hat{a})$ and with the RWA applied takes the form

$$\begin{aligned}\frac{\hat{H}}{\hbar} =& \frac{\epsilon}{2}\hat{\sigma}_z + \omega\hat{b}^\dagger\hat{b} + \omega_{\mathrm{r}}\hat{a}_{\mathrm{r}}^\dagger\hat{a}_{\mathrm{r}} + g\left(\hat{\sigma}_-\hat{b}^\dagger + \hat{\sigma}_+\hat{b}\right) + g_{\mathrm{r}}\left(\hat{\sigma}_-\hat{a}_{\mathrm{r}}^\dagger + \hat{\sigma}_+\hat{a}_{\mathrm{r}}\right) \\ &+ f\left(\hat{a}_{\mathrm{r}}\hat{b}^\dagger + \hat{a}_{\mathrm{r}}^\dagger\hat{b}\right) + \hat{\sigma}_x\eta_1\cos(\omega_1 t + \varphi_1) + \hat{\sigma}_x\eta_2\cos(\omega_2 t + \varphi_2).\end{aligned} \tag{25}$$

$g_{\mathrm{r}}/2\pi \sim 55\,\mathrm{MHz}$ denotes the coupling strength between qubit and readout resonator. The conjecture is verified by comparing classical master equation simulations with the photon exchange coupling f switched on and off, respectively, see Supplementary Figure 5(a). As visible in Supplementary Figure 5(b), the difference of both classical simulations (gray) follows the evolution of the bosonic mode population (red) in the rotating frame as obtained from the same simulation. The experimentally extracted bosonic mode population (blue) likewise agrees with the trend from the classical simulation. The photon exchange coupling assumes the form of a cross-Kerr interaction after diagonalization

in the subspace spanned by the bosonic mode and the readout resonator. We isolate this additional dispersive shift $\propto f^2$ by adding up measured data for the qubit prepared either in $|\mathrm{g}\rangle$ or $|\mathrm{e}\rangle$, as described in the Methods of the main text. Comparison with measured data suggests $f \sim$ MHz.

C. Effect of the qubit tunneling term in the effective Hamiltonian

For a realistic parasitic coupling $\eta_\mathrm{r} \sim 0.1\eta_1$ of the dominant Rabi drive to the bosonic mode and $\omega_\mathrm{eff}/2\pi = 5\,\mathrm{MHz}$, we obtain an effective qubit tunneling term

$$g\frac{\eta_\mathrm{r}}{2\omega_\mathrm{eff}}\hat{\sigma}_x \sim 2\pi \times 2.2\,\mathrm{MHz} \times \hat{\sigma}_x \sim 0.4\omega_\mathrm{eff} \times \hat{\sigma}_x, \tag{26}$$

by using the displaced effective Hamiltonian derived in Supplementary Equation (18). The thick black line in Supplementary Figure 6(a) shows a classical simulation of the qubit population according to the effective Hamiltonian, Supplementary Equation (12) without parasitic driving. For switching on the parasitic driving, $\eta_\mathrm{r} \neq 0$, the qubit is subject to a sub-rotation that however adheres with the envelope defined by the pure Hamiltonian. The dynamics of the bosonic mode is not qualitatively altered by the parasitic drive, as the displacement transformation defined in Supplementary Equation (16)

$$\hat{D}^\dagger \left(\omega_\mathrm{eff}\hat{b}^\dagger\hat{b} + \frac{1}{2}\eta_\mathrm{r}(\hat{b}^\dagger + \hat{b})\right)\hat{D} = \omega_\mathrm{eff}\hat{b}^\dagger\hat{b} + \mathrm{const.} \tag{27}$$

leaves the eigenenergies of the isolated harmonic oscillator unchanged. The time evolution of the Hamiltonian $\omega_\mathrm{eff}\hat{b}^\dagger\hat{b} + \frac{1}{2}\eta_\mathrm{r}(\hat{b}^\dagger + \hat{b})$ is depicted in Supplementary Figure 6(b) for varying η_r.

Supplementary Note 6. QUANTUM REVIVALS FOR INITIAL QUBIT STATE $|\mathrm{g}\rangle$, $|\mathrm{e}\rangle$

We demonstrate that the position of the quantum revival corresponds to $2\pi/\omega_\mathrm{eff}$ in quantum simulations with initial state prepared in $|0\rangle \otimes |\mathrm{g}\rangle$, where both the qubit and the bosonic mode are in its groundstate. Classical simulations are done for $\kappa \sim 3.9 \times 10^6\,\mathrm{s}^{-1}$ and $1/T_1 = 0.2 \times 10^6\,\mathrm{s}^{-1}$, $1/T_2 = 2.0 \times 10^6\,\mathrm{s}^{-1}$ and in the absence of a parasitic drive of the bosonic mode.

We find a better agreement of experimental data with classical simulations for a slightly increased geometric coupling $g/2\pi \sim 5.5\,\mathrm{MHz}$ as compared to the measured value in Fig. 2 in the main text of $g/2\pi = 4.3\,\mathrm{MHz}$. This can be explained by the slight excess population due to the parasitic coupling of the Rabi drives to the bosonic mode and the effect may be additionally enhanced by a population of higher transmon levels. Increased decay and decoherence rates, accounting for a changed environmental spectral density in the rotating frame, did not lead to the correct ratio of revival and idling amplitudes. Simulations using $g/2\pi = 4.3\,\mathrm{MHz}$ show results with a $\sim 30\%$ decrease in the maximum population of the bosonic mode with no qualitative consequences. With the measured geometric coupling we approach an USC regime with $g_\mathrm{eff}/\omega_\mathrm{eff} \sim 0.6$ for the experiment depicted in Supplementary Figure 7(d). With the slightly increased value for g_eff we reach a relative coupling ratio of 0.7. Measured values of g_eff and the effective value are summarized in Supplementary Table 2.

Supplementary Note 7. VERIFICATION OF THE PARAMETER CONSTRAINT FOR $\eta_2 \neq 0$

We verify the simulation data for the full quantum Rabi model presented in the main text by comparison to measured data with intentionally departing from the required parameter constraints. While an increase in revival amplitude and an increased amplitude of the fast oscillations for larger Δt is visible by comparing Supplementary Figure 8(a), (b) presented in the main text, these signatures vanish for violating the parameter conditions

$$\begin{aligned}\varphi_1 &= \varphi_2 \\ \eta_1 &= \omega_1 - \omega_2\end{aligned} \tag{28}$$

required by the simulation scheme. We chose $\varphi_1 \sim \varphi_2 + \pi$ but $\eta_1 = \omega_1 - \omega_2$ in Supplementary Figure 8(c) and $\omega_2 = \omega_1 - \eta_1 - 2\pi \times 10\,\mathrm{MHz}$ while $\varphi_1 = \varphi_2$ in Supplementary Figure 8(d) in order to demonstrate that the desired experimental features vanish.

An additional limitation of the simulation quality is imposed by an uncertainty of the effective Rabi frequency η_1, which is extracted from a rather broad peak in the Fourier transformed qubit evolution, see Supplementary Figure 5. This causes the main constraint of the simulation scheme $\eta_1 = \omega_1 - \omega_2$ to be poorly satisfied in particular at small simulation times.

Supplementary Note 8. SPECTROSCOPY OF THE AVOIDED CROSSING BETWEEN QUBIT AND BOSONIC MODE

The coupling between qubit and bosonic mode is pre-characterized in a two tone spectroscopy measurement using a vector network analyzer and a microwave source. The dispersive readout resonator shift is measured with a continuous microwave probe tone and an additional microwave drive tone is applied through the same transmission line to excite the qubit transition. The fit of the observed avoided crossing yields a minimum line separation of $2g/2\pi = 7.8\,\mathrm{MHz}$.

---

[1] D. Ballester, G. Romero, J. J. García-Ripoll, F. Deppe, and E. Solano, "Quantum simulation of the ultrastrong-coupling dynamics in circuit quantum electrodynamics," Phys. Rev. X **2**, 021007 (2012).

[2] A. Leggett, S. Chakravarty, A. Dorsey, M. Fisher, A. Garg, and W. Zwerger, "Dynamics of the dissipative two-state system," Rev. Mod. Phys. **59**, 1–85 (1987).

[3] E.T. Jaynes and F.W. Cummings, "Comparison of quantum and semiclassical radiation theories with application to the beam maser," Proc. IEEE **51**, 89–109 (1963).

[4] J. Braumüller, M. Sandberg, M. R. Vissers, A. Schneider, S. Schlör, L. Grünhaupt, H. Rotzinger, M. Marthaler, A. Lukashenko, A. Dieter, A. V. Ustinov, M. Weides, and D. P. Pappas, "Concentric transmon qubit featuring fast tunability and an anisotropic magnetic dipole moment," Appl. Phys. Lett. **108**, 032601 (2016).

[5] A. Lukashenko and A. V. Ustinov, "Improved powder filters for qubit measurements," Rev. Sci. Instrum. **79**, 014701 (2008).

[6] J.R. Johansson, P.D. Nation, and F. Nori, "Qutip: An open-source python framework for the dynamics of open quantum systems," Comp. Phys. Comm. **183**, 1760–1772 (2012).

[7] J.R. Johansson, P.D. Nation, and F. Nori, "Qutip 2: A python framework for the dynamics of open quantum systems," Comp. Phys. Comm. **184**, 1234–1240 (2013).

[8] J. Koch, T. M. Yu, J. Gambetta, A. A. Houck, D. I. Schuster, J. Majer, A. Blais, M. H. Devoret, S. M. Girvin, and R. J. Schoelkopf, "Charge-insensitive qubit design derived from the cooper pair box," Phys. Rev. A **76**, 042319 (2007).

[9] J. Braumüller, J. Cramer, S. Schlör, H. Rotzinger, L. Radtke, A. Lukashenko, P. Yang, S. T. Skacel, S. Probst, M. Marthaler, L. Guo, A. V. Ustinov, and M. Weides, "Multiphoton dressing of an anharmonic superconducting many-level quantum circuit," Phys. Rev. B **91**, 054523 (2015).

FIGURES

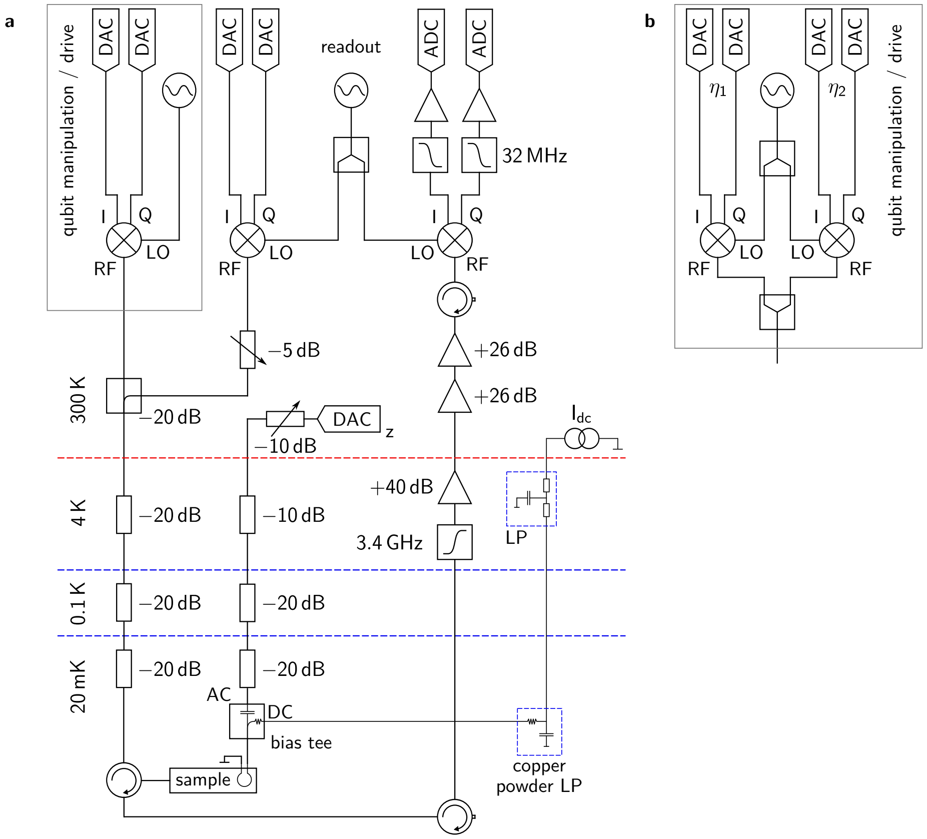

Supplementary Figure 1. **Schematic microwave setup used for the quantum simulation experiment** (a) Setup used for the experiments requiring only one Rabi drive tone. (b) Modification of the microwave setup (substituting the gray rectangle) for the application of two drive pulses with amplitudes η_1, η_2. The drive pulses are generated with two separate IQ mixers, sharing a common local oscillator (LO) input, and combined subsequently.

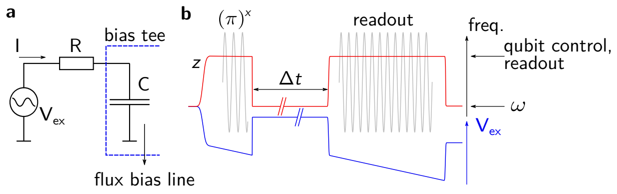

Supplementary Figure 2. **Compensation for finite time constant in the bias tee** (a) Simplified schematic circuit diagram prevailing the flux pulse generation. Microwave pulses of amplitude V_{ex} pass a resistor R and charge the capacitor which is part of the bias tee. (b) Due to the finite time constant τ of the bias tee, voltage pulses of amplitude V_{ex} following the blue line are applied to its input such that the resulting flux through the on chip flux coil follows the ideal pulse sequence depicted in red.

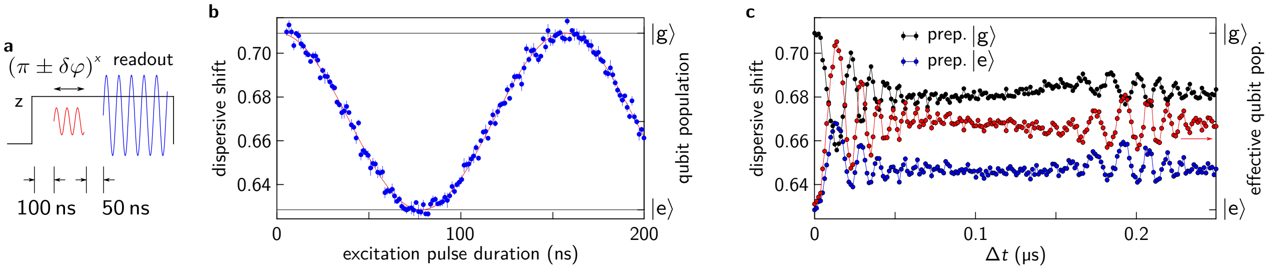

Supplementary Figure 3. **Calibration of the qubit basis** (a) Schematic pulse sequence used for the calibration. Qubit and bosonic mode are on resonance initially. A fast flux pulse is applied to tune the qubit out of resonance for excitation and readout. The length of the excitation pulse corresponding to the rotation angle φ around the Bloch sphere is varied during the calibration. (b) Qubit Rabi oscillations using the z pulsing sequence used in the simulation experiment and with a sinusoidal fit (red) applied. We identify the computational qubit basis spanned by the groundstate $|\mathrm{g}\rangle$ and the first excited state $|\mathrm{e}\rangle$. (c) Dispersive shift of the readout resonator for a simulation sequence with $\omega_{\mathrm{eff}}/2\pi = 5\,\mathrm{MHz}$ and the qubit prepared in $|\mathrm{e}\rangle$ (blue) or $|\mathrm{g}\rangle$ (black). The faintly visible bulge cancels by evaluating their difference, plotted in red. Error bars throughout the figure denote a statistical standard deviation as detailed in the Methods of the main text.

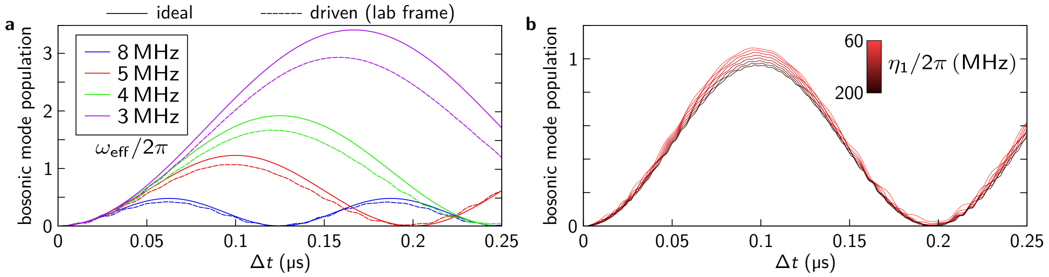

Supplementary Figure 4. **Verification of the simulation scheme for the ideal system** (a) Time evolution of the bosonic mode population for a simulation sequence with $\eta_2 = 0$. We compare its population in the ideal quantum Rabi Hamiltonian (solid line) with the population of the bosonic mode in the laboratory frame with the drive applied (dashed line). Despite the fact that an infinite energy reservoir is supplied by the drive, the population follows the expected one rather well. This remains also true for varying the drive amplitude η_1. Different colors correspond to a varying ω_{eff}. (b) Bosonic mode population for $\omega_{\mathrm{eff}}/2\pi = 5\,\mathrm{MHz}$ and a varying drive amplitude η_1. The evolution and maximum population is confirmed to be independent of η_1 in first order. Master equation simulations here are performed without taking into account dissipation and neglect parasitic driving of the bosonic mode.

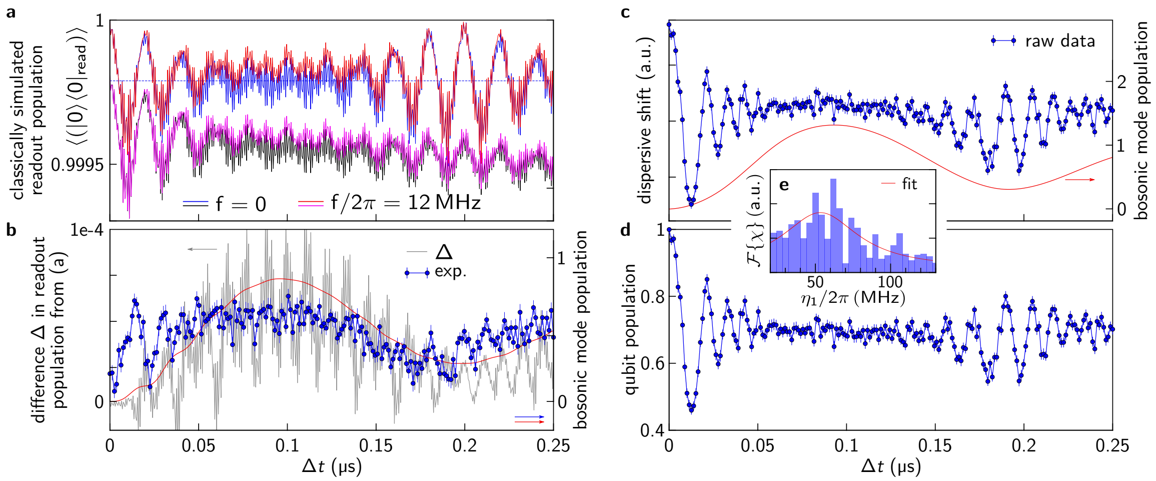

Supplementary Figure 5. **Verification of the photon exchange coupling between the bosonic mode and the readout resonator** (a) Classical master equation simulation of the vacuum projection $\left|0\right\rangle\left\langle 0\right|$ of the readout resonator state for vanishing photon exchange coupling $f = 0$ (blue) and for $f/2\pi = 12\,\text{MHz}$ (red), disregarding dissipation. The blue dashed line denotes the mean value of the $f = 0$ simulation as a guide to the eye. Magenta and black traces show the same classical simulations in the presence of dissipation and are shifted for better visibility. The additional bulge in the presence of the photon exchange coupling $f > 0$ is apparent. (b) The difference (gray) between both classical simulations from (a) including dissipation follows the experimentally measured bosonic mode population (blue). Both data sets are fitted to the time evolution of the bosonic mode population (red), as obtained from the same classical simulation. (c) Dispersive shift of the readout resonator including both the shifts induced by qubit and bosonic mode. The red line depicts the classically simulated population of the bosonic mode. (d) Extracted qubit signal in the original qubit basis after subtracting the additional shift induced by the bosonic mode, based on the classically simulated expectation depicted in (c). The qubit was prepared in $\left|e\right\rangle$. From Fourier transformation (e) we obtain $\eta_1/2\pi \sim 52\,\text{MHz}$. Error bars throughout the figure denote a statistical standard deviation as detailed in the Methods of the main text.

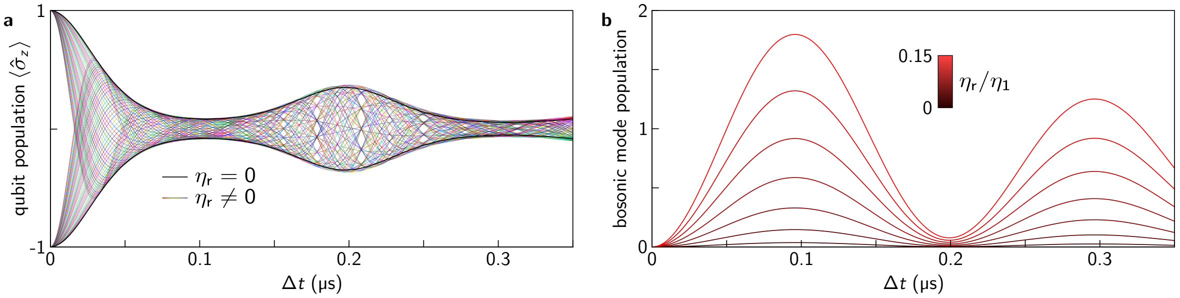

Supplementary Figure 6. **Qubit tunneling term in the effective Hamiltonian** (a) Classical simulation of the qubit population for the effective Hamiltonian, Eq.(18), without the qubit tunneling term $\eta_r = 0$ (black) in comparison with simulations for $\eta_r \neq 0$, plotted in colors. One can see that the additional qubit tunneling term $\propto \hat{\sigma}_x$ introduces a rotation while complying with the envelope of the ideal Hamiltonian, Supplementary Equation (12). We demonstrate this by plotting the evolution for various values of η_r and $\omega_{\text{eff}}/2\pi = 5\,\text{MHz}$. (b) Classically simulated bosonic mode population for a harmonic oscillator under static transversal drive. The periodicity of the evolution in the quantum Rabi model is reproduced up to a scaling factor.

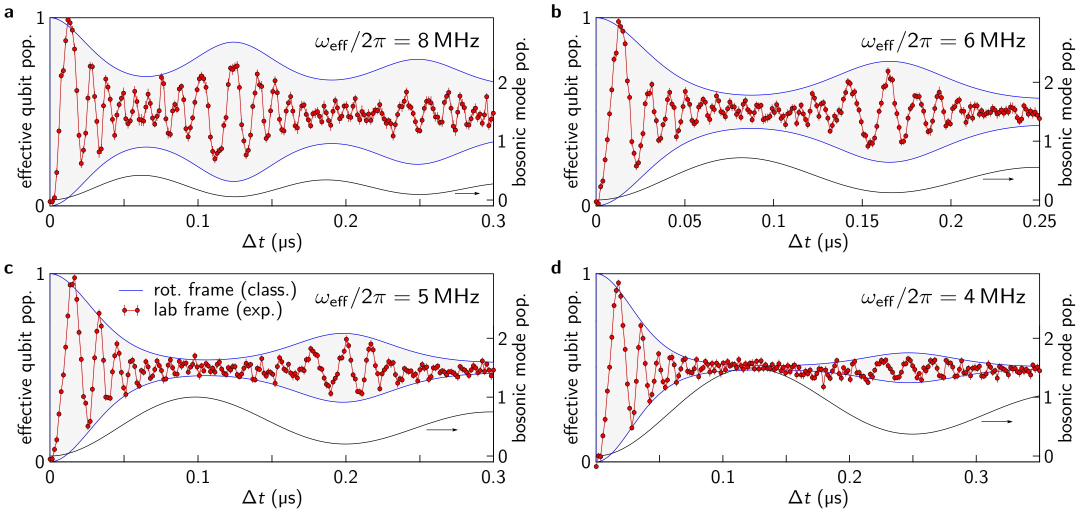

Supplementary Figure 7. **Quantum collapse and revival signatures for various** ω_{eff} The qubit is prepared in its ground-state $|g\rangle$ and the bosonic mode is initially in the vacuum state $|0\rangle$. The first revival appears at $2\pi/\omega_{eff}$, respectively, and the different plots correspond to a varying ω_{eff}. The blue line shows a classical master equation simulation of the ideal effective Hamiltonian in the rotating frame, while the red data points are the qubit population evolution in the effective qubit frame. The black line shows the classically simulated bosonic mode population in the rotating frame, which increases with decreasing ω_{eff}. Note that the scale on the horizontal axis is not equal for each plot. The depicted qubit signal is extracted from measured data with the protocol described in the Methods in the main text and based on the classical simulations (black) in the absence of a parasitic drive of the bosonic mode. Error bars denote a statistical standard deviation as detailed in the Methods of the main text.

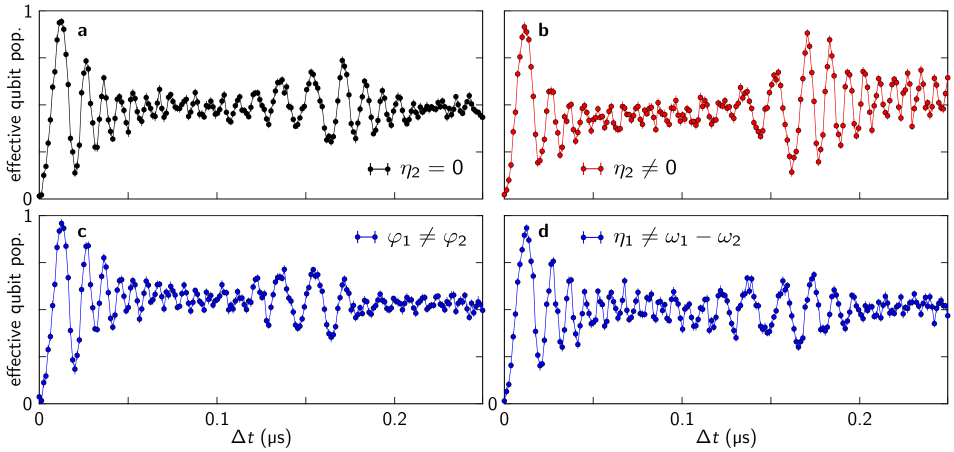

Supplementary Figure 8. **Verification of the parameter constraint for** $\eta_2 \neq 0$ Measurements for $\eta_2 = 0$ (a) and the proper parameter choice (b) when $\eta_2 \neq 0$ as shown in Fig. 4 in the main text with $\eta_1/2\pi = 58$ MHz. The measurement satisfying the required parameter constraints (b) is compared to measurements with parameters intentionally violating the phase matching condition, $\varphi_1 \neq \varphi_2$ (c) and the condition $\eta_1 \neq \omega_1 - \omega_2$ (d). When the required constraints are not satisfied, the expected signatures are suppressed. The dispersive shift of the readout resonator induced by the bosonic mode is subtracted based on classically simulated data. Error bars throughout the figure denote a statistical standard deviation as detailed in the Methods of the main text.

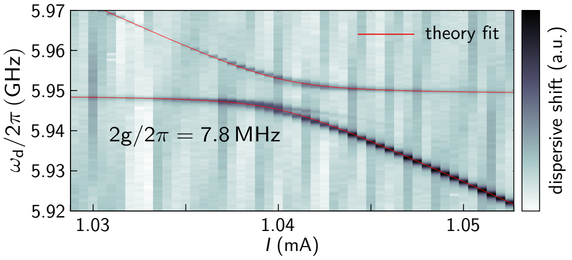

Supplementary Figure 9. **Avoided crossing between qubit and bosonic mode in spectroscopy** The qubit transition frequency is tuned by a dc current applied to the flux coil. The dispersive shift of the readout resonator is proportional to the excitation number of the qubit and is depicted in colors.

TABLES

$\omega/2\pi$	E_J/E_C	α/h	$1/T_1$	κ	$\omega_r/2\pi$	$\omega_{eff}/2\pi$	$\eta_1/2\pi$	$\eta_2/2\pi$
5.948 GHz	50	−0.36 GHz	$(0.2 \pm 0.12) \times 10^6\,s^{-1}$	$(3.9 \pm 0.14) \times 10^6\,s^{-1}$	8.86 GHz	< 8 MHz	∼ 50 MHz	∼ 3 MHz

Supplementary Table 1. **List of relevant device and simulation parameters.** Errors are extracted from the fit of the vacuum Rabi oscillations in 2 in the main text.

spectroscopy	vacuum Rabi	master eq. simulation
3.9 MHz	4.3 MHz	5.5 MHz

Supplementary Table 2. **Geometric coupling $g/2\pi$ between qubit and bosonic mode.** Values are obtained from the spectroscopic measurement in Supplementary Note 8, the vacuum Rabi experiment in Fig. 2 in the main text and the value used in master equation simulations.
